# The modern search for the Holy Grail: is neuroscience a solution?

**DOI:** 10.3389/fnhum.2014.00388

**Published:** 2014-06-04

**Authors:** Navot Naor, Aaron Ben-Ze'ev, Hadas Okon-Singer

**Affiliations:** ^1^Department of Psychology, University of HaifaHaifa, Israel; ^2^Department of Philosophy, University of HaifaHaifa, Israel

**Keywords:** happiness, hedonia, eudaimonia, fMRI, philosophy of neuroscience, neurophilosophy

## Abstract

Neuroscience has become prevalent in recent years; nevertheless, its value in the examination of psychological and philosophical phenomena is still a matter of debate. The examples reviewed here suggest that neuroscientific tools can be significant in the investigation of such complex phenomena. In this article, we argue that it is important to study concepts that do not have a clear characterization and emphasize the role of neuroscience in this quest for knowledge. The data reviewed here suggest that neuroscience may (1) enrich our knowledge; (2) outline the nature of an explanation; and (3) lead to substantial empirical and theoretical discoveries. To that end, we review work on hedonia and eudaimonia in the fields of neuroscience, psychology, and philosophy. These studies demonstrate the importance of neuroscientific tools in the investigation of phenomena that are difficult to define using other methods.

*“Begin discussion—by saying what is happiness.” Charles Darwin*.

A note in one of Darwin's early workbooks, written 2 years following his return from the voyage on board the H. M. S. Beagle and in the midst of writing an account of this voyage (McMahon, 2006, p. 410).

## Introduction: neuroscience and the study of the humanities

As scientists we seek to make sense of the world. Yet as Socrates lamented, the more we learn, the more we learn how little we know. This paradoxical condition makes one wonder how we can make sense of that which we can barely even characterize. Such is the case in the study of consciousness and free will (Gazzaniga, 2006), of God, faith and morality (Dawkins, 2006; Haidt, 2008; Kauffman, 2010), and of emotions (Ben-Ze'ev, 2000; Griffiths, 2001). As brain imaging evolved, the belief that these new scientific tools can help us solve old humanistic problems evolved alongside it. The strongest supporters of these tools argue that all we need to do to solve philosophy's greatest unanswered questions is to reconstruct them as questions of neuroscience. The fiercest objectors, conversely, exclaim that neuroscience is nothing more than a set of meaningless correlations, “neuro-nonsense” that adds nothing to the explanation of the human condition (Scruton, 2012). As Mencken (1949) said, “There is always an easy solution to every human problem—neat, plausible, and wrong.” In our view, such is the case for both of the aforementioned views.

**Figure 1:**
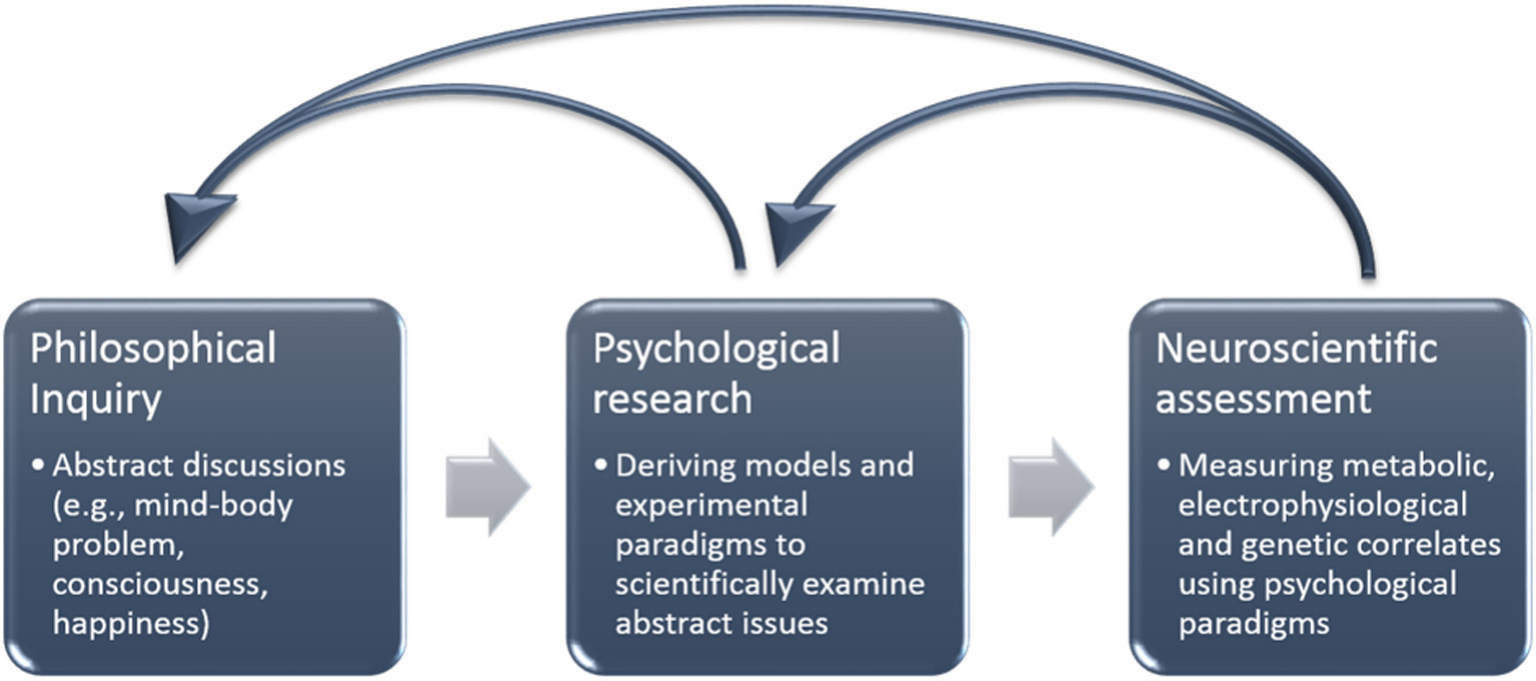
**A schematic model demonstrating the outflow from abstract philosophical concepts to psychological paradigms and neuroscientific methods of investigation**. Neuroscientific findings can enhance our understanding of both psychological and philosophical inquiries.

In this article we focus on the study of happiness as an example of a concept whose characterization is debateable. We discuss the advantages of studying concepts that do not have a clear characterization, and emphasize the role of neuroscience in this inquiry. We argue that neuroscience may (1) enrich our knowledge about the underlying physiological correlates of human behavior; (2) outline and characterize the nature of human experience, refute contradictory explanations and expand existing theories; and (3) lead to substantial empirical and theoretical discoveries. Indeed, neuroscience may reveal findings hitherto inaccessible through other disciplines and subsequently result in substantial theoretical developments (Figure 1).

## Studying emotions: is definition necessary for scientific investigation?

By their nature, emotions are complex phenomena, affected by various cultural, contextual, and personal features. Therefore, it has been claimed that a single definition cannot capture the variance and essence of emotions (Ben-Ze'ev, 2000; Griffiths, 2001). Ben-Ze'ev (2000) believes that emotional complexity stems primarily from the following factors: (1) emotions are highly sensitive to contextual and personal factors; (2) emotions do not appear in isolation, but rather within a cluster of emotional attitudes; and (3) the linguistic use of emotional terms is confusing. Coping with this complexity requires adopting adequate conceptual tools. Three such tools are the following: (1) prototype categories having neither clear-cut boundaries nor a unitary degree of membership; (2) the use of various levels of descriptions, such as neuroscience, biology, psychology, sociology, and philosophy, and of various perspectives within each level; and (3) the use of systematic classifications of different emotional aspects, such as systematically discerning the basic evaluative and motivational aspects of emotions (Ben-Ze'ev, 2000).

Specifically, in the following we show how a philosophical notion, in our case the Aristotelian notion of eudaimonia, is examined by means of psychology and then by neuroscience. These connections are easier to explain than the direct connection between neuroscience and philosophy, although we do not reject the possibility of such a direct connection.

## What is happiness?

**Figure 2:**
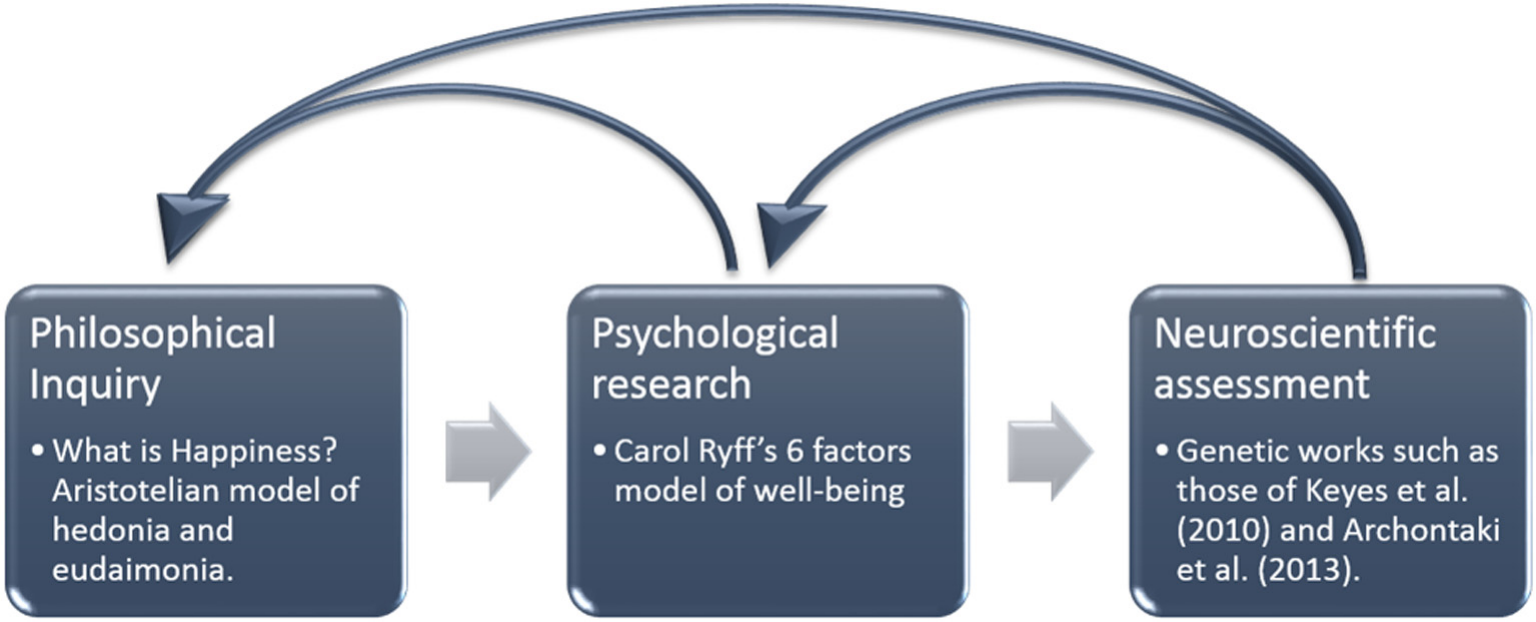
**Happiness as an example of the way in which a neuroscientific approach may shed light on longstanding philosophical debates**.

Happiness has been the subject of centuries of philosophical and psychological scrutiny. We examine the central Aristotelian notion of eudaimonia from a philosophical perspective and then describe it in psychological terms. After that we examine the relevance of neuroscience to these discussions (Figure 2).

Aristotle distinguishes between hedonia, which expresses the feeling aspect of happiness, and eudaimonia, which expresses the more general notion of human prosperity and sense of well-being. Whereas eudaimonia refers to quality of life as a whole, and especially to an individual's virtuous functioning in life, hedonia refers merely to having good feelings, or getting what you want, or enjoying something you are doing. Hedonia is typically a feature of eudaimonia. Nevertheless, although feeling good about life and functioning in life are related, they are distinct phenomena (Deci and Ryan, 2008; Keyes and Annas, 2009). Whereas hedonia can be found among grazing animals, both hedonia, and eudaimonia are found in humans.

The concept of eudaimonia is relevant to discussions concerning various philosophical issues, among them the meaningfulness of life (Baumeister et al., 2013), the nature of mental health (Keyes, 2002), and the nature of love (Mendham, 2007). Examining this notion on both the psychological and the neuroscientific levels of descriptions may contribute to clarification of the Aristotelian notion and to its revision in light of empirical evidence.

The notion of hedonia is simpler and easier to measure, while the notion of eudaimonia is more complex and harder to quantify. Whereas hedonia refers to a present subjective state, eudaimonia connects the present, past and future in an individual's virtuous activities, which are expressions of the individual's unique nature and capacities. These differences are reflected in the greater number of scientific articles devoted to hedonia (Kringelbach and Berridge, 2009). For example, a recent PubMed search using the term “hedonia” yielded 51 papers, while a similar search using the term “eudaimonia” yielded only 15 papers.

Kashdan et al. (2008) assert that the distinction between hedonic and eudaimonic well-being is unwarranted philosophically and scientifically. They argue that the two types of happiness are not easily segregated and that eudaimonia is not well-defined and has not been measured consistently. They further claim that empirical evidence currently suggests that hedonic and eudaimonic well-being overlap conceptually, and may represent psychological mechanisms that operate together. Their criticism is refuted by the psychologists Carol Ryff (2013) and Alan Waterman (2008), the sociologist Corey Keyes and the philosopher Julia Annas (Keyes and Annas, 2009). The dispute's very presence indicates the usefulness of discussions between philosophers, psychologists, and sociologists on philosophical issues. Neuroscientists, we believe, can successfully contribute to this discussion.

## The use of neuroscientific methods in the investigation of happiness

Hedonia is not merely the sensation we feel as a result of a pleasing stimulation. Rather, it is the outcome of specific neural activation (referred to as “hedonic hotspots”) toward such stimulation, without it even the most pleasing sensation would have no effect (Berridge and Kringelbach, 2011). These regions include the nucleus accumbens shell, the ventral pallidum, the forebrain, and the limbic-cortical and deep brainstem regions, including the parabrachial pontine nucleus (Peciña et al., 2006). Hedonia, as Aristotle noted, is by no means a unique human experience. In fact one might be surprised by the cross specie similarity in the brain regions that govern hedonia's genesis. In fact a mapping of the archipelago of hedonic hotspots in rodent's brain using drug micro-injections revealed a picture analogs to the one found in humans (Berridge and Kringelbach, 2011). Furthermore, both the mid-lateral orbitofrontal cortex and the medial orbitofrontal edge have been implicated in coding of hedonic experiences (Berridge and Kringelbach, 2011). These two areas however, have very distinct roles. Activity in the mid-lateral orbitofrontal cortex correlates with subjective pleasantness of stimuli such as food, drugs, and music. This activity acts as a sort of pleasure barometer, tracking changes in subjective pleasure from sensation, as in the inconceivable case of eating chocolate to the point of satiety (Berridge and Kringelbach, 2011, 2013). On the other hand, the activity of the medial orbitofrontal edge codes the subjective valence of positive and negative events, and does not seem to change as the pleasantness of a sensation diminishes (Berridge and Kringelbach, 2011), thus its response to chocolate will always outshine the one for broccoli. This immunization to change may serve as the gateway between neural regions involved in hedonia and those related to the vaguer concept of eudaimonia. These neuroimaging studies enhance our understanding of the manner in which an experience gloss coating is done by a network of hedonic hotspots. Moreover, they serve as critical milestones in refining how hedonia is characterized as they support the view that hedonia and eudaimonia do not overlap, at least not neurologically. Lastly, as proposed by Berridge and Kringelbach (2011), they bring us a step closer to understanding that hedonic balance might be the key for a generation of eudaimonia and well-being.

As mentioned, not many studies have examined eudaimonia. What turned the tides was Ryff's model of well-being (1989, 2013), which bridged between philosophical discussions and neuroscientific studies. Ryff's model drew on the roots of the Aristotelian concept, underlining the central role of having a purpose to life, autonomy, personal growth, environmental mastery, positive relationships, and self-acceptance (Archontaki et al., 2013). Ryff cemented her model by supporting it with the work of some of the most prominent thinkers and psychologists of the 20th century, among them Jung, Maslow, Rogers, and Erikson. Without the development of Ryff's quantitative tools, the solemnity of the philosophical accounts would have very little relevance to our daily life, and therefore would not have counted for much.

Recently, Keyes et al. (2010) sought to tap into the genetic basis for eudaimonia. In a twin-based study they inspected the structure of both genetic and environmental influences on eudaimonia and found a strong hereditable genetic factor influencing eudaimonia. The factors that comprise eudaimonia are the subject of an interesting debate. While Ryff suggested a six-factor model of eudaimonia comprising autonomy, personal growth, self-acceptance, purpose in life, positive relations with others, and environmental mastery, others have claimed that the overlap between these factors is so high that they constitute a single factor (Springer et al., 2006). To test these opposing theoretical views, Archontaki et al. (2013) employed a classic twin study approach. The six-factor well-being questionnaire (Ryff, 1989) was completed by 837 identical or fraternal twins. Structural equation modeling examined the impact of genetic and environmental factors on each well-being factor. The results support the view that eudaimonia is impacted by a complex genetic structure. Five distinct genetic factors were found to influence psychological well-being, manifesting biological commonalities between different sub-scales as well as unique aspects. Environmental effects were not supported by the data. Lewis et al. (2014) further found a positive association between the size of the right insular cortex, a region previously negatively associated with depression, and scores on Ryff's well-being scale. These results were found for the general score and for three independent subscales: purpose in life, positive relations, and personal growth. This association, however, is not an indication of casualty. Hence, we cannot know whether greater gray matter volume is a prerequisite for higher eudaimonia or a result of it.

Coltheart (2006, 2013; Tressoldi et al., 2012) noted that most neuroimaging studies focus on the localization of regions involved in certain cognitive and psychological functions. Notably, this suggestion is based on the (yet, disputed) belief that psychological processes can be localized in principle. Coltheart (2006) emphasized the need to use neuroimaging in order to distinguish between competing psychological theories. Perhaps the use of neuroimaging to inquire philosophically-complex concepts can further enhance our theoretic understanding of such concepts: although the experiments of Berridge and Kringelbach aim at localization, they are in line with the aim of distinguishing between competing theories, in the sense that the findings enhance our understanding of hedonia as opposed to eudaimonia. Had the researchers obtained similar activations for both processes, the conclusions might have been different. Similarly, the genetic studies mentioned above are in line with this aim, although it should be noted that while Coltheart focused on neuroimaging, here we have adapted his framework to other scientific tools. In line with our initial argument about the necessity for characterization as a basis for scientific inquiry, these studies demonstrate the feasibility, as well as the importance, of examining complex and yet-to-be-defined concepts. Furthermore, these examples demonstrate the dynamic reciprocal interaction between philosophical and psychological views and models on the one hand and biological-based investigations on the other. Given a theoretical model as an anchor, biological tools can be used to further our knowledge and fine-tune our psychological views.

## A neuroscientific view on traditional philosophical questions

As noted, recent studies have used neuroscientific methods and tools, including neuroimaging, genetics, event-related potentials, behavioral reaction time, and accuracy measures, to explore the underlying mechanisms of happiness. In recent years the use of neuroimaging has become common in many fields. For example, the use of neuroscientific approaches has been debated in the context of consciousness (Block, 2007; Koch and Tsuchiya, 2007). Similar to emotions, consciousness has been the subject of research in philosophy for centuries. Ever since Descartes proposed that “Cogito ergo sum”—I think, therefore I am—the worlds of philosophy and science have spiraled into an ongoing debate about the dualism of body and mind (e.g., Damasio, 1994). Recent neuroimaging studies of patients in a vegetative state have revealed neural activation in regions known to be involved in face processing when these patients were presented with pictures depicting emotional facial expressions (Sharon et al., 2013). These findings have provided important insights about information processing at different levels of consciousness, which could not have been provided by other methods. Furthermore, neuroimaging and electrophysiology studies comparing stimuli presented in conscious and non-conscious manners have provided insights about the neural underpinnings of conscious and non-conscious information processing (see Haynes, 2009 and Rees, 2013, for reviews). What is relevant here is that consciousness is not a clear and defined phenomenon. On the contrary, as Gazzaniga (2012) stated in a recent interview, there is still a long road to travel before science is able to define what consciousness is or understand how it works. Nevertheless, it is important to investigate different aspects of consciousness using neuroscientific, philosophical, and biological methods in order to shed light on the experiences of awareness and of the self.

More generally, other concepts that are merely characterized but not defined, such as emotion, should nevertheless be the subject of scientific inquiry. For example, Theodoridis and Nelson (2012) discuss the promises and pitfalls of neuroimaging in the context of political psychology, and conclude that neuroimaging should be considered a valid tool for studying political psychology (see also Hruby, 2012, for a recommendation to employ neuroscience in education studies).

These examples demonstrate how neuroscientific methods illuminate important aspects of complex phenomena. We argue that neuroscientific investigation can provide new insights that may lead to theoretical changes by revealing findings that were not hypothesized a priori. For example, Siman-Tov et al. (2007) set out to compare the processing of emotional and neutral face stimuli. To that end, they presented face pictures in the right and the left visual fields. Serendipitously, following left visual field stimulation they found enhanced activations in attention-related brain regions. They further conducted a causal analysis to explain the left field bias, leading them to propose a model that differs from the classic model of attention bias (see also follow-up investigation in Okon-Singer et al., 2011).

It is interesting to note that neuroscience, and particularly neuroimaging, has led to great expectations, as well as to the opposite view that such methods cannot contribute to the study of psychological phenomena (e.g., Coltheart, 2006; Page, 2006; Poldrack, 2006; Papanicolaou, 2007; Aue et al., 2009). Tressoldi et al. (2012) argue that most neuroimaging studies have focused on localizing cognitive processes, and thus have not resulted in new insights regarding psychological theories. Other researchers have pointed to different improper uses of fMRI. Vul et al. (2009) claimed that very high correlations found in fMRI studies are due to non-independent analyses. Aue et al. (2009) noted that studies which infer that regional brain activation reflects a specific psychological process without considering other factors that could have led to similar activation have resulted in general skepticism regarding the advantage of using fMRI. Yet despite criticism, the common and important conclusion is that neuroimaging, when properly used, is a valid tool to investigate psychological phenomena.

## Conclusion

Developments in the field of neuroscience have brought with them the concomitant evolution of two new branches of philosophy. One is neuro-philosophy, or the use of neuroscientific tools for examining philosophical questions. The other is related to the philosophy of neuroscience, or the use of philosophical inquiries in neuroscientific practices (Bickle et al., 2012). In this inquiry of happiness we used both of these with the hope of formulating the fullest account of this thorny subject.

Today's neuroscientist can say virtually nothing about the psychological content of an emotion merely by looking at the coloring of this or that region of the brain. Instead, the neuroscientist needs the conceptual analysis of the philosopher and the behavioral explanation of the psychologist, which in turn can be used and manipulated in the lab. In the same manner philosophers must take into account the work of psychologists and neuroscientists and refine their understanding accordingly. These three can no longer exist and operate in separate realms. Rather, only through a joint effort can we truly formulate a complete and accurate understanding of human behavior.

As for happiness, by no means is the quest for its characterization a purely philosophical endeavor. Enlightenment thinkers were the first to promise human beings happiness in the here and now. By the time the dust settled on both great revolutions of that era, the French and the American, it was widely accepted that “happiness of all” is a “self-evident truth” (Declaration of the Rights of Man and of the Citizen; Declaration of Independence, respectively). The only problem was that while we were all entitled to pursue happiness, we barely knew what we were pursuing, sending humanity off on a wild goose chase. It should be of little surprise, then, that after decades of pursuing unattainable happiness, depression has become the second leading cause of disability worldwide (Ferrari et al., 2013).

Be that as it may, the future is not so bleak. We are in the midst of the neuroscience revolution, in which ever improving technologies and methods are transforming the human ability to understand the brain and to intervene with its functioning (Wolpe, 2002). It would seem, however, that neuroscience has an unexpected way of alleviating pain and bettering lives, not only through the exploration of new drugs and therapeutic methodologies. Rather, when right questions are asked, tools used properly, and data interpreted accurately, neuroscience can help characterize what has otherwise been undefinable, as in the case of happiness.

### Conflict of interest statement

The authors declare that the research was conducted in the absence of any commercial or financial relationships that could be construed as a potential conflict of interest.
