# Erratum on: The modern search for the Holy Grail: is neuroscience a solution?

**DOI:** 10.3389/fnhum.2014.00515

**Published:** 2014-07-16

**Authors:** Frontiers Production Office

**Affiliations:** Frontiers Production OfficeLausanne, Switzerland

**Keywords:** happiness, hedonia, eudaimonia, fMRI, philosophy of neuroscience, neurophilosophy

Due to a production error, in the published article the second and third authors got replaced. This error does not change the scientific conclusions of the article in any way. The publisher apologizes for this mistake and the correct order appears hereafter:

Correct author order: Navot Naor, Aaron Ben-Ze'ev, Hadas Okon-Singer.

The original article has been updated.

## Conflict of interest statement

The authors declare that the research was conducted in the absence of any commercial or financial relationships that could be construed as a potential conflict of interest.
